# Cost-effectiveness of active monitoring versus antidepressants for major depression in primary health care: a 12-month non-randomized controlled trial (INFAP study)

**DOI:** 10.1186/s12888-015-0448-3

**Published:** 2015-03-31

**Authors:** Maria Rubio-Valera, Imma Beneitez, María Teresa Peñarrubia-María, Juan V Luciano, Juan M Mendive, Paul McCrone, Martin Knapp, Ramon Sabés-Figuera, Katarzyna Kocyan, Javier García-Campayo, Antoni Serrano-Blanco

**Affiliations:** Fundació Sant Joan de Déu, Esplugues de Llobregat, Spain; Primary Care Prevention and Health Promotion Research Network (RedIAPP), Barcelona, Spain; School of Pharmacy, Universitat de Barcelona, Barcelona, Spain; Open University of Catalonia (UOC), Barcelona, Spain; Primary Care Health Centre Bartomeu Fabrés Anglada, Servei d’Atenció Primària Delta Llobregat, Àmbit Costa de Ponent, Institut Català de la Salut, Gavà, Spain; La Mina Primary Care Centre, Institut Català de la Salut, Sant Adrià de Besós, Barcelona, Spain; Institute of Psychiatry, King’s College London, De Crespigny Park, London, UK; Personal Social Services Research Unit, London School of Economics and Political Science, Houghton Street, London, UK; Institute for Prospective Technological Studies/Joint Research Centre, European Commission, Sevilla, Spain; Miguel Servet Hospital, University of Zaragoza, Instituto Aragones de Ciencias de la Salud, Zaragoza, Spain; Parc Sanitari Sant Joan de Déu, Sant Boi de Llobregat, Spain

**Keywords:** Depressive Disorder, Antidepressive Agents, Active Monitoring, Watchful Waiting, Costs and Cost Analysis, Primary Health Care

## Abstract

**Background:**

Clinical practice guidelines for the treatment of major depressive disorder (MDD) recommend antidepressants for patients with moderate-severe depression and active monitoring for patients with mild-moderate symptoms. The feasibility and efficiency of active monitoring has not been proven conclusively. The aim of this study is to evaluate the cost-effectiveness of active monitoring in comparison to antidepressants for primary care patients with mild-moderate MDD.

**Methods/Design:**

This is a 12-month follow-up multicenter observational prospective controlled trial. Patients are enrolled in 12 primary care centers in Barcelona (Spain). Eligible patients are adults (≥18 years-old) with a new episode of MDD that sign a written consent to participate. This is a naturalistic study in which general practitioners (GPs) use their professional judgment to allocate patients into active monitoring or antidepressants groups. GPs treat the patients following their clinical criteria. At baseline, GPs complete a questionnaire (sociodemographic/job characteristics, training, attitude towards depression, interest on mental health and participation in communication groups). Patients’ measurements take place at baseline and after six and 12 months. Main outcome measures include severity of depression (PHQ-9), health-related quality of life (EuroQol-5D) and use of healthcare and social care services (Client Service Receipt Inventory). Secondary outcomes include diagnosis of MDD according to DSM-IV diagnostic criteria (SCID-I), disability (WHO-DAS), anxiety (BAI), comorbidities, medication side-effects and beliefs about medicines (BMQ).

The analysis will be done according to the intention to treat analysis. Missing data will be imputed using multiple imputation by chained equations. To minimize the bias resulting from the lack of randomization, a propensity score will be used. Incremental effects and costs between groups will be modelled in each of the imputed databases using multivariate generalized linear models and then combined as per Rubin’s rules. Propensity scores will be used to adjust the models. Incremental cost-effectiveness ratios will be calculated by dividing the difference in costs between groups by the difference in effects. To deal with the uncertainty, resampling techniques with bootstrapping will be used and cost-effectiveness planes and cost-effectiveness acceptability curves will be constructed. A series of sensitivity analyses will be performed.

**Discussion:**

Given the high burden and costs generated by depressive disorder, it is important that general practitioners treat major depression efficiently. Recent evidence has suggested that antidepressants have low benefits for patients with mild to moderate major depression. For such cases of depression, active monitoring exists as a treatment option, but it is not without difficulties for implementation and its effectiveness and efficiency have not been demonstrated conclusively. The results of the study will provide information on which is the most efficient approach to treat patients with mild to moderate major depression in primary care.

**Trial registration:**

ClinicalTrials.gov: NCT02245373

## Background

Major depressive disorder (MDD) generates high expenditure that is caused largely by its high prevalence, its recurrence and chronicity and because it usually affects young people of working age [1,2]. The social cost of this mental disorder is also very high, given the risk of suicide and the serious negative impacts in social, occupational and family situations [3]. From the work carried out by Goldberg and Huxley in the eighties, it is widely recognized that major depression is mainly treated at the primary care level [4]. More recent studies indicate that 10% to 16% of primary care patients fulfill criteria for a diagnosis of MDD [5,6]. It is therefore important that general practitioners (GPs) be capable of detecting and treating MDD using evidence-based medicine and taking the particularities of the primary level of care into account.

Psychotropic drugs, mostly antidepressants and anxiolytics, are highly prescribed in primary care in our setting as they are the standard treatment for MDD. About 70% of patients with a mood disorder receive psychotropic drugs in Catalan primary care [7]. Moreover, almost 25% of patients who do not fulfil criteria for a mood or anxiety disorder are also prescribed a psychotropic drug [7]. This percentage could include patients with minor depression or psychological distress that the GP misdiagnosed [8,9]. A review showed that benefits of antidepressants depend on the severity of the depressive episode, which may be minimal in patients with mild to moderate symptoms [10]. Another review showed that brief psychological therapy (brief cognitive behavioral therapy (CBT), counselling and problem solving therapy) were effective treatments in primary care for patients with anxiety, depression and mixed mental health problems [11]. The recommendations of the clinical practice guidelines for the treatment of MDD vary according to the severity of the case. The guidelines recommend medication for patients with moderate and severe depression and active monitoring (or watchful waiting) and referral for further assessment and interventions for those with mild to moderate symptoms [12,13].

Active monitoring has been described as a decision “between the clinician and the patient to not treat the condition and to intermittently reassess its status along some rational time course in follow-up” [14]. According to the Catalan guideline [13], for patients receiving active monitoring, the GP should monitor the patient within 15 days of the first visit. Other recommendations are to accompany closed monitoring with low intensity psychosocial therapy (e.g. problem-solving techniques, counselling or brief CBT), individual guided self-help programs based on CBT or structured and supervised exercise programs of moderate intensity. The guidelines only recommend the use of antidepressants in mild major depression if the patients have a history of moderate or severe episodes of depression or if they have other medical conditions or associated comorbidities.

Despite being recommended in guidelines, the effectiveness of active monitoring has not been proven conclusively. There has been a study showing a low likelihood of spontaneous remission in minor depression following a one-month active monitoring period in primary care. However, the same study showed that engaging in regular pleasant activities conferred an advantage to remission in minor depression.. The study recommended the use of feasible interventions that promote activity and decrease avoidant coping styles in primary care [14]. Furthermore, an economic evaluation of usual GP care with or without antidepressant medication for patients with minor or mild-major depression observed no differences in cost-effectiveness between the two treatment strategies, recommendeding that GPs sparingly prescribe antidepressants to mildly depressed patients [15]. On the contrary, in older primary care patients with dysthymia and minor depression, a problem-solving behavioral based psychotherapy in primary care showed smaller benefits for depressive symptoms than paroxetine [16]. More recently, the THREAD study compared selective serotonin reuptake inhibitors (SSRIs) plus supportive care versus supportive care alone for mild to moderate depression with somatic symptoms in primary care [17]. The study showed that adding an antidepressant was more cost-effective than supportive care alone.

An additional problem regarding active monitoring is the lack of consensus on what is the best strategy for implementation and how to overcome the lack of adherence to recommendations. A study examining the factors associated with GPs’ choice of watchful waiting to care management of depression showed barriers that included the clinicians lack of knowledge on psychotherapy and the lack of availability of mental health professionals for referral [18]. In order to choose watchful waiting, GPs may be able to schedule follow-up appointments with the patient and perform low intensity psychosocial interventions or have available referral services for the patient. However, GPs do not routinely receive training in psychological therapy and the adequate number of follow-up sessions (one within the first 15 days and from six to eight during the 10–12 weeks following the diagnosis [13]) can be unrealistic given the existent burden in primary care. This is illustrated by the study by Hegel and colleagues, where the proportion of patients that received at least one contact with the GP during the first month of watchful waiting was of only 21% [14].

It is still necessary to show whether non-pharmacological interventions in mild-moderate major depression can be a feasible and cost-effective alternative compared to pharmacological interventions in actual practice primary care. The aim of this study is to evaluate the cost-effectiveness of active monitoring in comparison to the use of antidepressants for the treatment of mild to moderate MDD in primary care usual practice in a 12-month non-randomized controlled trial.

## Methods

We followed the SPIRIT statement for reporting trial protocols [19]. This is the first published version of the protocol.

### Study design

This is a 12 month follow-up multicenter observational prospective controlled trial comparing patients that receive antidepressant drugs with those who do not. The study was approved by the Clinical Research Ethics Commitee of the Sant Joan de Déu Foundation (CEIC Fundació SJD; Reference Number: EPA-24-12) and the Clinical Research Ethics Commitee of The Jordi Gol i Gurina Foundation (CEIC IDIAP; Reference Number: 5013 – 002).

### Study setting and GP enrolment

GP enrolment was conducted six months before patient recruitment. GPs from the province of Barcelona were invited to participate. The University Institute in Primary Care Research Jordi Gol (IDIAP Jordi Gol), which gives technical support to every professional that works in Primary Care in the Catalan Public Health System, spread the invitation to participate in the study to all the GPs from the province of Barcelona. Furthermore, the research team contacted the Primary Care Centers with whom they had worked in previous research studies to invite them to participate.

The study is conducted in 12 primary care centers in the province of Barcelona (Spain). The participating centers have between six and 17 primary care teams (each of them consisting of a GP and a nurse) and attend to a population of 250,000 to 350,000 inhabitants. Sixty-eight GPs participated in the recruitment of patients for the study.

Prior to the study, GPs received a three hour-training on the study protocol, diagnostic criteria for depression, and national guidelines for the treatment (pharmacological and non-pharmacological) of MDD in primary care, divided into two 1.5-hour sessions. Session 1: Diagnosis and non-pharmacological treatment for MDD (active monitoring, sleep hygiene, counseling, frequency of follow-up visits, health education and low intensity psychological therapies); and Session 2: pharmacological treatment of MDD. During the study, a monthly newsletter is sent to the participating GPs to remind them of the topics presented in the training seminars and to inform them about the study progress.

At the beginning of the study, the GPs completed a questionnaire collecting the following variables: sociodemographic characteristics, job characteristics, training, attitude towards depression, interest on mental health and participation in communication groups [20].

### Eligibility criteria and recruitment

Eligible patients are adults (≥18 years-old) who receive a diagnosis for a new episode of MDD. The following patients are excluded: those that have taken an antidepressant medication in the previous 60 days; those presenting psychotic or bipolar disorders or on antipsychotics, lithium or antiepileptics in the previous six months; those with history of drug abuse or dependency; those with cognitive impairment that prevents an assessment interview; and those who refuse to give signed consent to participate.

GPs recruit patients for the study from their daily list of patients attending the practice until they reach five patients for each group (active monitoring or pharmacological treatment). Maximum recruiting time is 12 months. For patients meeting the inclusion criteria, GPs inform them of the study’s aim and procedures during the medical visit, where a written informed consent is also obtained. GPs then refer patients for their first assessment appointment.

### Interventions

This is a naturalistic study. GPs use their professional clinical judgment to recommend a treatment option to the patient. GPs can recommend a non-pharmacological intervention (Active Monitoring Group) or a pharmacological treatment with antidepressants (Medication Group) following their own clinical criteria and experience.

The patients in the Active Monitoring Group receive the usual treatment that the GPs perform when applying active monitoring without a pharmacological treatment. According to the Catalan guideline [13], which has been presented to all the GPs, active monitoring requires a first follow-up visit within the following 15 days. Afterwards, it recommends from six to eight follow-up visits over 10–12 weeks, where the GPs can consider low intensity psychosocial therapies such as counseling, problem-solving techniques or on-line CBT. Also, it recommends structured and supervised exercise programs of moderate intensity. As part of the stepped care model, in case the patient’s condition does not improve, the GP can intensify the treatment and initiate antidepressants.

Adherence to active monitoring is controlled through patient interviews (patients are asked the number of control visits with the GP and the recommendations to deal with depression from their GP). Also, at the end of the study, the GPs will be asked to describe the actions that were taken with patients in the Active Monitoring Group.

The patients in the Medication Group recieve the antidepressants usually prescribed in Spanish primary care at doses usually recommended according to their symptoms and characteristics. The national guidelines recommend initiating a pharmacological treatment with SSRIs (particularly with citalopram, sertraline, paroxetine or fluoxetine) in accordance with the Catalan Health Service’s recommendations following cost-effectiveness criteria [13].

Adherence to antidepressants is monitored through two methods: pharmacy records (computerized pharmacy records that register information about medication including active principle, dose and units supplied in the patient’s clinical history at the time of purchase), and patients’ self-reported adherence (with the 4-item scale developed by Morisky and colleagues [21]).

At the moment of patient inclusion, GPs complete a form that includes the following information: patient allocation (active monitoring or antidepressants) and reasons for the allocation and type and dose of antidepressant prescribed, if any. Withdrawal or changes in treatment and the reasons (initiation of antidepressants or changes in active principle) will be registered in another form. This will allow the evolution of treatment in naturalistic conditions to be monitored.

### Outcomes and participant timeline

**Figure 1:**
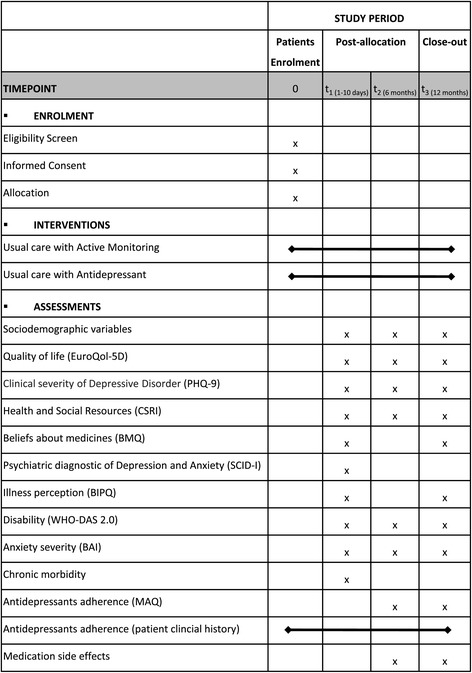
**Schedule of enrolment, interventions and assessments.** PHQ-9 = Patient Health Questionnaire; CSRI = Client Service Receipt Inventory; BMQ = Beliefs about Medicines Questionnaire; SCID-I = Structured Clinical Interview for DSM Disorders; BIPQ = Brief Illness Perception Questionnaire; WHO-DAS 2.0 = World Health Organization Disability Assessment Schedule; BAI = Beck Anxiety Inventory; MAQ = Medication Adherence Questionnaire.

The primary outcome of the study is cost-effectiveness, measured in terms of incremental cost per reduction of the severity of depression achieved and in incremental costs per quality adjusted life years (QALYs) gained. Figure 1 shows all the measures administered at each assessment visit as well as the time schedule of patients.

Costs are collected from a societal perspective. Use of health care resources and lost productivity are assessed using the Client Service Receipt Inventory (CSRI) [22] with a recall period of 12 months at baseline and six months at point two and close-out. We collect information on productivity losses, health tests, hospital care (emergency visits and stays), secondary care (visits to psychologists, psychiatrist and other specialists), primary care (visits to GP and nurse), medication use and social care services (visits to social worker). Information on the use of psychotropic medicines (active principle, dose and units supplied) is also collected from computerized pharmacy records.

The unit costs of public healthcare services are obtained from the Official Bulletin of the Catalan Government. Costs of privately funded services are obtained from published tariffs. The mean price per milligram of active principle is calculated using the prices of the generic versions of all the presentations as reported in the Spanish Vademecum. Productivity losses will be calculated based on the human capital approach using information on the minimum and average daily wage in Spain (INE) [23].

Changes in the severity of depression are assessed using the Patient Health Questionnaire 9-item depression module (PHQ-9) [24-26]. The PHQ-9 is a nine-item scale with items scored from 0 (not at all) to 3 (nearly every day) on nine symptoms of depression. Summed scores range from 0 (no depressive symptoms) to 27 (all symptoms occurring daily). Summed scores of 20 to 27 correspond to severe symptoms; 15 to 19 to moderately severe; 10 to 14 to moderate symptoms; 5 to 9 to mild symptoms; and 0 to 4 to minimal symptoms.

The Spanish version of the EuroQol-5D (EQ-5D) is used to measure health-related quality of life [27-29]. The EQ-5D records self-reported problems in five domains (mobility, selfcare, usual activities, pain/discomfort and anxiety/depression) divided into three levels of severity (no problems, some problems, and extreme problems), thus generating 245 possible health states [30]. Each state corresponds to a single index value referred to as the tariff. Value 1.000 is the best health state and value 0.000 corresponds to being dead. The second part records the subject’s self-assessed health on a Visual Analogue Scale (VAS) on which the best and worst imaginable health states score 100 and 0, respectively. QALYs are calculated by multiplying the utility with the amount of time a patient spent in a particular health state. Linear interpolation is used for transitions between health states.

Clinical diagnosis according to DSM-IV diagnostic criteria was confirmed using the research version of the Structured Clinical Interview for DSM-IV Axis I Disorders (SCID-I) [31]. The modules of mood and anxiety disorders were used. A low concordance between diagnosis of MDD by GPs and SCID-I criteria has been described [9]. It was considered important to check the diagnosis with SCID-I. However, the study was naturalistic so the diagnostic according to DSM-IV criteria was informative and not used as an inclusion criteria. GPs were blind to the DSM-IV diagnosis and patient inclusion was performed according to their usual practice.

Disability was assessed using the 12-item interviewer administered version of the World Health Organization Disability Assessment Schedule (12-item WHO-DAS 2.0). Respondents have to indicate the level of difficulty experienced taking into consideration how they usually do the activity, including the use of any assistive devices and/or the help of a person. In each item, individuals have to estimate the level of disability during the previous month using a 5-point scale (none = 1, mild = 2, moderate = 3, severe = 4, extreme/cannot do = 5). The total score is calculated with a syntax provided by the WHO can range from 0 to 100 with higher scores reflecting greater disability. In the ERASMAP study, the Spanish version demonstrated adequate psychometric properties (Cronbach’s α = 0.89) and evidence of unidimensionality [32-34].

The Beck Anxiety Inventory (BAI) is a twenty-one item self-report inventory that evaluates the severity of anxiety. Each question has four possible answer choices that go from 0 (not at all) to 3 (severely) with total scores ranging from 0 (minimal level of anxiety) to 63 (severe anxiety) [35,36].

Chronic physical conditions were assessed using a “yes” or “no” check-list.

Medication Side effects: Evident side-effects are assessed using a brief check-list considering the most common side-effects of antidepressants. For each side-effect the intensity, frequency and causal relation with antidepressant drugs is assessed.

The Beliefs about Medicine Questionnaire (BMQ) assesses the cognitive representations of medication [37,38]. It has two sections, the BMQ-General and the BMQ-Specific. The BMQ-General evaluates general medication beliefs and comprises two 4-item factors: *General-Harm* (medicines are harmful, addictive or poisonous) and *General-Overuse* (medicines are overused or excessively trusted by doctors). The BMQ-Specific evaluates representations of specific medication prescribed for the patient, in this case antidepressants. This part was only administered to patients on the antidepressants group. The BMQ-Specific comprises two 5-item factors: *Specific-Necessity* (the need of antidepressants) and *Specific-Concerns* (dangers of use of antidepressants).

Sociodemographic characteristics are evaluated at the beginning of the study: age, gender marital status, education and working status.

### Statistical analysis

The analysis will be done according to the intention to treat analysis (all the patients will be included in the analysis in the group to which they were allocated independently of the treatment they finally received.

### Missing data

Missing data patterns will be evaluated to assess if it is plausible that data is missing at random [39]. To minimize bias resulting from the loss of information not following a completely at random reason, missing values will be imputed using multiple imputation by chained equations. The imputation model will include relevant socio-demographic and prognostic variables associated with the drop-outs and outcome variables and variables to be included in the final cost-effectiveness models [40].

### Propensity score calculation

The allocation of patients is done according to the GP’s decisions. Thus, it is expected to have bias, as groups could not be comparable. To minimize the bias resulting from the lack of randomization, we will use a propensity score. First, it will be evaluated where the probability of receiving active monitoring or antidepressants is affected by GP factors (socio-demographic characteristics, job characteristics, training, attitude towards depression, interest on mental health and participation in communication groups) and/or patient factors (socio-demographic characteristics, baseline severity of depression, presence of major depression according to SCID-I criteria, severity of anxiety, comorbid conditions and beliefs about medicines). Second, a logistic regression model will be used to calculate a propensity score. The dependent variable of this model will be the group (active monitoring or antidepressants) and the independent variables will be those that are associated with a higher or lower probability of receiving active monitoring or antidepressants.

### Incremental cost-effectiveness ratios calculation

Incremental effects and costs between groups will be modelled using generalized linear models. First, different distribution families and link functions will be tested and Akaike and Bayesian information criterion (AIC and BIC) will be used to decide the model that best fits the distribution of the effects (QALYs and severity of depression) and costs. Second, to select adjustment variables, socio-demographic and baseline clinical variables considered to be relevant will be tested in the models using likelihood ratio tests. All the models will be adjusted for gender, age and the propensity score. Difference in costs and effects will be calculated using the final models in each of the imputed databases and combined as per Rubin’s rules [40]. Incremental cost-effectiveness ratios will be calculated by diving the difference in costs between groups by the difference in effects.

### Cost-effectiveness planes and cost-effectiveness acceptability curves

To deal with the uncertainty in the sampling distribution of the incremental cost-effectiveness ratio, resampling techniques with bootstrapping will be used. Replications will be done in each of the imputed databases and then combined. A minimum of 5,000 replications will be generated. Bias-corrected and accelerated (BCa) confidence intervals will be estimated on each of the imputed databases and then averaged [41]. Bootstrapped pairs of cost and effect differences will be plotted on cost-effectiveness planes and used to construct the cost-effectiveness acceptability curves.

### Sensitivity analyses

At minimum, the following sensitivity analyses will be performed: 1) an analysis from the healthcare perspective; 2) a per protocol analysis; 3) an analysis using the mean average salary instead of the minimum average salary for productivity losses; 4) an analysis not adjusted for the propensity score; 5) an analysis including only the patients that fulfill DSM-IV criteria for MDD.

### Sample size

In cost-effectiveness studies, sample size calculations have been criticized and their usefulness has been questioned [42]. This calculation is influenced by many parameters, some of them related to costs, which must be specified a priori. However, knowledge about costs and deviations is scarce so it is required to make assumptions that affect the calculation of the sample size. Moreover, this calculation requires to decide, in advance, what the maximum acceptable incremental cost-effectiveness ratio or maximum willingness to pay will be. Again, where to put the cutoff points for these parameters is a complicated decision. Finally, it should be borne in mind that the results of the economic evaluation will be presented in cost-effectiveness acceptability curves. These curves are not based on statistical inference so the meaning of sample size calculations could be questioned again.

On the other hand, to deal with the uncertainty in the sampling distribution of the incremental cost-effectiveness ratio, it will be necessary to perform resampling techniques that require a minimum sample size. Previous experience in these randomized studies with naturalistic conditions indicates that a total of 150 patients per arm will suffice [41,43].

## Discussion

The results of this study will provide evidence about whether active monitoring in mild-moderate MDD can be a feasible and cost-effective alternative to pharmacological interventions in actual practice primary care. Although clinical practice guidelines recommend active monitoring, evidence of its effectiveness and efficiency is scarce and contradictory. This study will improve knowledge on active monitoring efficiency and, as far as is known, it will be the first study on this topic to be conducted in Spain. If non-pharmacological interventions would prove to be more cost-effective than pharmacological ones, this would show that preventing the use of antidepressants in patients with mild-moderate MDD could save the health care system money and protect patients from side-effects of non-necessary drugs.

The study has a series of limitations that must be considered. The naturalistic nature of the study implies the use of wide inclusion criteria and non-randomized group assignment, which increase the external validity of the results. However, wide inclusion criteria can increase the inter-subject variability and reduce the ability to detect differences. It is more likely for the GP to allocate patients presenting with mild depression to the active monitoring group than to the pharmacological treatment group. Conversely, for patients with moderate-severe depression, GPs might be more likely to choose pharmacological options than active monitoring. Propensity score techniques will be used to try to minimize the impact of this bias in the results of the study.

One limitation of the study is the GP selection. The group of GPs that decided to participate in the study could have a greater interest in mental health. This might include specific attitudes of those GPs when facing physiological problems in primary care and might not represent average GP behavior. Along these lines, it is possible in this study that GPs enroll patients that they think that will be more willing to participate and remain in the study. Also, all the health centers are located in the same health area, so the intervention can be biased for specific practices associated to local culture or characteristics of the health system.

Due to the burden of GPs, their lack of training on brief psychotherapy techniques and the lack of referral services available, a risk exists for active monitoring to become no treatment at all. For example, in a trial considering a one-month watchful waiting period only 21% of the sample had at least one contact with the physician during the first month. To guarantee naturalistic conditions in the present study, GPs received brief training on active monitoring principles. Interventions received by patients in the active monitoring group will be registered to determine what active monitoring really means in Catalan primary care real practice. Yet, non-adherence to antidepressants in our context is high [44,45]. A sensitivity analysis using a per protocol strategy of analysis will be performed to determine the impact of the adherence to the protocol both in the active monitoring and antidepressant groups.

Another limitation could be that the GPs introduce changes to the intervention (e.g. initiate antidepressants for patients in the active monitoring group or change active principle/dose in patients on antidepressants) without filling in the study’s registration. An attempt to minimize this will be made through monthly reminders to the GPs. Also, the patient’s history will be reviewed to gather information on medication.

## References

[1] Salvador-Carulla L, Bendeck M, Fernández A, Alberti C, Sabes-Figuera R, Molina C (2011). Costs of depression in Catalonia (Spain). J Affect Disord.

[2] Demyttenaere K, Bruffaerts R, Posada-Villa J, Gasquet I, Kovess V, Lepine JP (2004). Prevalence, severity, and unmet need for treatment of mental disorders in the World Health Organization World Mental Health Surveys. JAMA.

[3] Ustün TB, Ayuso-Mateos JL, Chatterji S, Mathers C, Murray CJL (2004). Global burden of depressive disorders in the year 2000. Br J Psychiatry.

[4] Golberg D, Huxley P (1992). Common Mental Disorders: a Biosocial Model.

[5] Serrano-Blanco A, Palao DJ, Luciano JV, Pinto-Meza A, Luján L, Fernández A (2010). Prevalence of mental disorders in primary care: Results from the diagnosis and treatment of mental disorders in primary care study (DASMAP). Soc Psychiatry Psychiatr Epidemiol.

[6] King M, Nazareth I, Levy G, Walker C, Morris R, Weich S (2008). Prevalence of common mental disorders in general practice attendees across Europe. Br J Psychiatry.

[7] Rubio-valera M, Fernández A, Luciano JV, Hughes CM, Pinto-meza A, Moreno-küstner B (2012). Psychotropic prescribing in catalonia: Results from an epidemiological study. Fam Pract.

[8] Fernández A, Rubio-Valera M, Bellón JA, Pinto-Meza A, Luciano JV, Mendive JM (2012). Recognition of anxiety disorders by the general practitioner: results from the DASMAP study. Gen Hosp Psychiatry.

[9] Fernández A, Pinto-Meza A, Bellón JA, Roura-Poch P, Haro JM, Autonell J (2010). Is major depression adequately diagnosed and treated by general practitioners? Results from an epidemiological study. Gen Hosp Psychiatry.

[10] Fournier JC, DeRubeis RJ, Hollon SD, Dimidjian S, Amsterdam JD, Shelton RC (2010). Antidepressant drug effects and depression severity: a patient-level meta-analysis. JAMA.

[11] Cape J, Whittington C, Buszewicz M, Wallace P, Underwood L (2010). Brief psychological therapies for anxiety and depression in primary care: meta-analysis and meta-regression. BMC Med.

[12] National Institute for Clinical Excellence.Depression: Management of Depression in Primary and Secondary Care; 2009.

[13] Agència d’Informació A i Q en SP director de salut mental i addiccions. D de SG de C (2010). Guía de Pràctica Clínica catalana: Adaptació al model sanitari català de la guia de pràctica clínica sobre el maneig de la depressió major en l’adult.

[14] Hegel MT, Oxman TE, Hull JG, Swain K, Swick H (2006). Watchful waiting for minor depression in primary care: remission rates and predictors of improvement. Gen Hosp Psychiatry.

[15] Bosmans JE, Hermens MLM, de Bruijne MC, van Hout HPJ, Terluin B, Bouter LM (2008). Cost-effectiveness of usual general practitioner care with or without antidepressant medication for patients with minor or mild-major depression. J Affect Disord.

[16] Williams JW, Barrett J, Oxman T, Frank E, Katon W, Sullivan M (2000). Treatment of Dysthymia and Minor Depression in Primary Care: A Randomized Controlled Trial in Older Adults.

[17] Kendrick T, Chatwin J, Dowrick C, Tylee A, Morriss R, Peveler R (2009). Randomised controlled trial to determine the clinical effectiveness and cost-effectiveness of selective serotonin reuptake inhibitors plus supportive care, versus supportive care alone, for mild to moderate depression with somatic symptoms in primary care. Health Technol Assess.

[18] Meredith LS, Cheng WJY, Hickey SC, Dwight-Johnson M (2007). Factors associated with primary care clinicians’ choice of a watchful waiting approach to managing depression. Psychiatr Serv.

[19] Chan AW, Tetzlaff JM, Altman DG, Laupacis A, Gøtzsche PC, Krleža-Jerić K (2013). SPIRIT 2013 statement: Defining standard protocol items for clinical trials. Ann Intern Med.

[20] Mira JJ, Llinás G, Gil V, Orozco D, Palazón I, Vitaller J (1998). Validation of an instrument for identifying styles of the professional practice of the primary care doctor. Aten Primaria.

[21] Morisky DE, Green LW, Levine DM (1986). Concurrent and predictive validity of a self-reported measure of medication adherence. Med Care.

[22] Chisholm D, Knapp MRJ, Knudsen HC, Amaddeo A, Gaite L, Van Wijngaarden B. Client socio-demographic and service receipt inventory - European version: Development of an instrument for international research. EPSILON study 5. Br J Psychiatry. 2000;177:s28-33. 10.1192/bjp.177.39.s28 10945075

[23] Spanish National Institute of Statistics: Minimum wage survey; 2015.

[24] Spitzer RL (1999). Kroenke K, Williams JB: Validation and utility of a self-report version of PRIME-MD: the PHQ primary care study. Primary Care Evaluation of Mental Disorders. Patient Health Questionnaire. JAMA.

[25] Diez-Quevedo C, Rangil T, Sanchez-Planell L, Kroenke K, Spitzer RL. Validation and utility of the patient health questionnaire in diagnosing mental disorders in 1003 general hospital Spanish inpatients. Psychosom Med. 2001;63:679–86. 10.1097/00006842-200107000-00021 11485122

[26] Kroenke K, Spitzer RL, Williams JBW (2001). The PHQ-9: Validity of a brief depression severity measure. J Gen Intern Med.

[27] Group TE (1990). a new facility for the measurement of health-related quality of life. Heal Policy.

[28] Badia X, Schiaffino A, Alonso J, Herdman M (1998). Using the EuroQol 5-D in the Catalan general population: Feasibility and construct validity. Qual Life Res.

[29] Badia X, Roset M, Montserrat S, Herdman M, Segura A (1999). [The Spanish version of EuroQol: a description and its applications. European Quality of Life scale]. Med Clin (Barc).

[30] Dolan P, Gudex C, Kind P, Williams A. A social tariff for EuroQol: results from a UK general population survey. York: Centre for Health Economics, University of York; 1995.

[31] First MB et, Spitzer RL, Gibbon M, Williams JBW. Structured Clinical Interview for DSM-IV Axis I Disorders, Clinician Version (SCID-CV). 1997:132.

[32] Luciano JV, Ayuso-Mateos JL, Fernandez A, Aguado J, Serrano-Blanco A, Roca M (2010). Utility of the twelve-item World Health Organization disability assessment schedule II (WHO-DAS II) for discriminating depression “caseness” and severity in Spanish primary care patients. Qual Life Res.

[33] Luciano JV, Ayuso-Mateos JL, Fernández A, Serrano-Blanco A, Roca M, Haro JM (2010). Psychometric properties of the twelve item World Health Organization Disability Assessment Schedule II (WHO-DAS II) in Spanish primary care patients with a first major depressive episode. J Affect Disord.

[34] Luciano JV, Ayuso-Mateos JL, Aguado J, Fernandez A, Serrano-Blanco A, Roca M (2010). The 12-item World Health Organization Disability Assessment Schedule II (WHO-DAS II): a nonparametric item response analysis. BMC Med Res Methodol.

[35] Sanz Fernández J, Navarro ME. Propiedades psicométricas de una versión española del Inventario de Ansiedad de Beck (BAI) en estudiantes universitarios. Ansiedad y estrés. 2003;9:59–84.

[36] Beck AT, Epstein N, Brown G, Steer RA (1988). An inventory for measuring clinical anxiety: psychometric properties. J Consult Clin Psychol.

[37] Horne R, Weinman J, Hankins M (1999). The beliefs about medicines questionnaire: The development and evaluation of a new method for assessing the cognitive representation of medication. Psychol Health.

[38] Beléndez Vázquez M, Hernández Mijares A, Horne R, Weinman J (2007). Evaluación de las creencias sobre el tratamiento: validez y fiabilidad de la versión española del Beliefs about Medicines Questionnaire.

[39] Sterne JAC, White IR, Carlin JB, Spratt M, Royston P, Kenward MG (2009). Multiple imputation for missing data in epidemiological and clinical research: potential and pitfalls. Br Med J.

[40] White IR, Royston P, Wood AM (2011). Multiple imputation using chained equations: Issues and guidance for practice. Stat Med.

[41] Rubio-Valera M, Bosmans J, Fernández A, Peñarrubia-María M, March M, Travé P (2013). Cost-effectiveness of a community pharmacist intervention in patients with depression: a randomized controlled trial (PRODEFAR Study). PLoS One.

[42] Al MJ, van Hout BA, Michel BC, Rutten FF (1998). Sample size calculation in economic evaluations. Health Econ.

[43] Serrano-Blanco A, Pinto-Meza A, Suárez D, Peñarrubia MT, Haro JM (2006). Cost-utility of selective serotonin reuptake inhibitors for depression in primary care in Catalonia. Acta Psychiatr Scand Suppl.

[44] Rubio-Valera M, March Pujol M, Fernández A, Peñarrubia-María MT, Travé P, López del Hoyo Y (2013). Evaluation of a pharmacist intervention on patients initiating pharmacological treatment for depression: A randomized controlled superiority trial. Eur Neuropsychopharmacol.

[45] Serna MC, Cruz I, Real J, Gascó E, Galván L (2010). Duration and adherence of antidepressant treatment (2003 to 2007) based on prescription database. Eur Psychiatry.

